# Casein kinase II–dependent phosphorylation of DNA topoisomerase II suppresses the effect of a catalytic topo II inhibitor, ICRF-193, in fission yeast

**DOI:** 10.1074/jbc.RA118.004955

**Published:** 2019-01-11

**Authors:** Norihiko Nakazawa, Orie Arakawa, Masahiro Ebe, Mitsuhiro Yanagida

**Affiliations:** From the G0 Cell Unit, Okinawa Institute of Science and Technology Graduate University, Onna-son, Okinawa 904-0495, Japan

**Keywords:** DNA topoisomerase, protein phosphorylation, chromosomes, yeast genetics, mitosis, cell cycle, anticancer drug, casein kinase II, chromosome segregation, DNA topoisomerase II, fission yeast, ICRF-193

## Abstract

DNA topoisomerase II (topo II) regulates the topological state of DNA and is necessary for DNA replication, transcription, and chromosome segregation. Topo II has essential functions in cell proliferation and therefore is a critical target of anticancer drugs. In this study, using Phos-tag SDS-PAGE analysis in fission yeast (*Schizosaccharomyces pombe*), we identified casein kinase II (Cka1/CKII)–dependent phosphorylation at the C-terminal residues Ser^1363^ and Ser^1364^ in topo II. We found that this phosphorylation decreases the inhibitory effect of an anticancer catalytic inhibitor of topo II, ICRF-193, on mitosis. Consistent with the constitutive activity of Cka1/CKII, Ser^1363^ and Ser^1364^ phosphorylation of topo II was stably maintained throughout the cell cycle. We demonstrate that ICRF-193–induced chromosomal mis-segregation is further exacerbated in two temperature-sensitive mutants, *cka1–372* and *cka1/orb5-19*, of the catalytic subunit of CKII or in the topo II nonphosphorylatable alanine double mutant *top2-S1363A,S1364A* but not in cells of the phosphomimetic glutamate double mutant *top2-S1363E,S1364E*. Our results suggest that Ser^1363^ and Ser^1364^ in topo II are targeted by Cka1/CKII kinase and that their phosphorylation facilitates topo II ATPase activity in the N-terminal region, which regulates protein turnover on chromosome DNA. Because CKII-mediated phosphorylation of the topo II C-terminal domain appears to be evolutionarily conserved, including in humans, we propose that attenuation of CKII-controlled topo II phosphorylation along with catalytic topo II inhibition may promote anticancer effects.

## Introduction

DNA topoisomerase II (topo II)^3^ passes dsDNA through the other dsDNA by transiently breaking and rejoining the strand (1–3). In eukaryotic cells, topo II relaxes supercoiled DNA and catalyzes catenation–decatenation, as well as knotting–unknotting of circular DNA. The supercoil relaxation activity of topo II is crucial for resolving negatively supercoiled DNA, which forms behind replication or transcription machinery, and also positively supercoiled DNA, which forms in front of it. In mitosis, proper separation of replicated sister chromatids requires topo II decatenation activity (4–6). Using *Schizosaccharomyces pombe top2* temperature-sensitive (ts) and cold-sensitive (cs) mutants, it has been proposed that topo II continuously acts on chromosomes to disentangle topologically intertwined sister chromatids throughout mitosis (7–10).

Topo II is a critical target for anticancer chemotherapy because of its essential role in cell proliferation. Two types of topo II inhibitor are widely known as anticancer drugs (11–13). The best known topo II–targeting drugs are etoposide and doxorubicin, which inhibit the rejoining step after cleaving one of the dsDNA strands (14). These drugs induce DNA double-stranded breakage and are thus termed “topo II poisons.” In contrast, “catalytic inhibitors” block ATPase activity prerequisite for dsDNA release from the DNA–topo II covalent complex after DNA rejoining (11). ICRF-193, a bisdioxopiperazine derivative (meso-4,4-(2,3-butanediyl)-bis (2,6-piperazinedione)), is a catalytic topo II inhibitor (15, 16). This drug causes mitotic chromosomal mis-segregation but does not interfere with DNA replication, resulting in a ploidy increase in mammalian cells (17–19). In fission yeast, ICRF-193 inhibits mitotic chromosomal segregation accompanied by unique spindle dynamics, leading to a ploidy increase (20). Thus, the effect of anticancer catalytic topo II inhibitors on mitotic chromosomes and spindle dynamics is validated and conserved from yeast to human cells.

**Figure 1.**
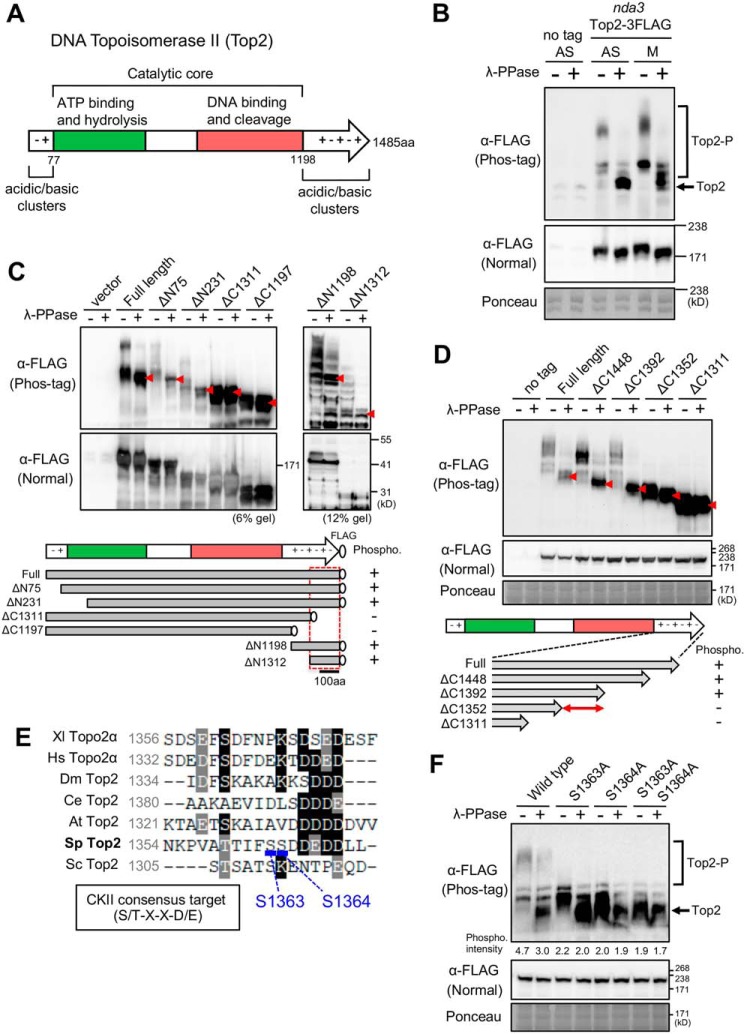
**Phos-tag analyses reveal that DNA Top2 Ser^1363^ and Ser^1364^ in the C-terminal charged region are phosphorylated.** *A*, schematic of the fission yeast Top2 polypeptide. Top2 consists of a catalytic core and acidic/basic clusters at both ends of the protein. The catalytic core contains two evolutionarily conserved domains, an ATP-binding hydrolytic domain (*green*), and a DNA-binding cleavage domain (*red*). *B*, Phos-tag analysis of FLAG-tagged Top2 protein. Extracts of a cs *nda3-KM311* β-tubulin mutant strain expressing Top2–3FLAG were prepared from asynchronously cultured (*AS*) and mitotically arrested cells (*M*) and run on SDS-PAGE gels in the presence or absence of 25 μm Phos-tag. The untagged strain was used as a negative control. Each sample was preincubated with phage λ PPase (+) or buffer (−). Ponceau staining served as a loading control. Anti-FLAG antibodies detected phosphorylated Top2 proteins (*Top2-P*) in asynchronous and mitotically arrested cells. The position of the marker band is not indicated in the phos-tag blot because the marker proteins were also highly phosphorylated, and precise positions were unclear. *C*, Phos-tag analysis of truncated Top2 proteins. *Top panel*, N- or C-terminally truncated Top2-FLAG proteins were mildly overproduced under the inducible nmt promoter (plasmid Rep81) in the absence of thiamine in WT *S. pombe* cells. A strain containing only the vector was used as a control. Positions of unphosphorylated bands are indicated by *red arrowheads. Bottom panel*, truncated fragments and their phosphorylation (*Phospho*) status are indicated (+, phosphorylated; −, unphosphorylated). A *red dashed box* shows the predicted region of phosphorylation. *aa*, amino acids. *D*, C-terminally truncated Top2 proteins were expressed under the native promoter with the chromosomally integrated FLAG-tagged gene. Top2 C terminus phosphorylation is predicted between residues 1352 and 1391 (*double-headed arrow* in the *bottom panel*). *E*, the amino acid sequence around Ser^1363^ and Ser^1364^ of *S. pombe* Top2, with seven-amino-acid sequences. The consensus target sequence for CKII is shown. *Xl*, *Xenopus laevis*; *Hs*, *Homo sapiens*; *Dm*, *Drosophila melanogaster*; *Ce*, *Caenorhabditis elegans*; *At*, *Arabidopsis thaliana*; *Sp*, *Schizosaccharomyces pombe*; *Sc*, *Saccharomyces cerevisiae. F*, the Phos-tag–mediated mobility shift of the Top2 protein disappeared as a result of alanine substitutions for Ser^1363^ and Ser^1364^. The intensity of the smeary phosphorylated Top2 bands was quantified and is indicated relative to the background.

Topo II is a phosphoprotein, and its evolutionarily divergent C-terminal domain (CTD) is preferentially phosphorylated in budding yeast, fission yeast, *Drosophila*, and human cells (21–28). In budding yeast and human cultured cells, casein kinase II (CKII) phosphorylates the topo II CTD (26, 29). The topo II CTD has been thought to be dispensable for enzymatic activity itself, acting as a regulator of topo II through nuclear localization or chromosomal targeting of topo II proteins (30–32). In *S. pombe*, papain digestion of topo II protein produces an ∼30-kDa C-terminal hydrophilic domain in which multiple phosphorylation sites have been predicted (Fig. 1*A*) (33). The physiological significance of topo II CTD phosphorylation in this organism has been unclear because topo II dephosphorylated by phosphatase treatment is still enzymatically active (25).

In this study, we identified CKII-dependent phosphorylation sites in the CTD of *S. pombe* topo II protein using Phos-tag SDS-PAGE analysis and specific anti-phospho antibodies. We present evidence that phosphorylation at these sites diminishes the effect of an anticancer catalytic topo II inhibitor, ICRF-193, on mitotic chromosome segregation.

## Results

### Phos-tag analysis of S. pombe DNA Topoisomerase II (Top2) identified residues Ser^1363^ and Ser^1364^ as phosphorylation sites in the Top2 C-terminal region

To detect phosphorylation of *S. pombe* Top2, we first performed Phos-tag analysis to identify phosphorylated protein bands in SDS-PAGE by decreasing the mobility of phosphoproteins (34). We used the Top2–3FLAG strain, which contains a chromosomally integrated 3FLAG-tagged *top2*^+^ gene under the native promoter (35). Cell extracts were prepared from asynchronously cultured (33 °C, 70–80% of cells are in interphase) or mitotically arrested (20 °C) β-tubulin cs mutant *nda3-KM311* cells. As shown in immunoblot patterns using anti-FLAG antibodies (Fig. 1*B*), lower-mobility bands of Top2–3FLAG were produced in the presence of Phos-tag (Fig. 1*B*, *top panel*) in both asynchronously cultured and mitotic cells, whose mobility increases after treatment with λ protein phosphatase (PPase). Mitotic phosphorylation of Top2–3FLAG was slightly increased compared with that in asynchronous cells. These results confirmed that *S. pombe* Top2 is a phosphoprotein (25).

To determine which subdomain of Top2 protein is phosphorylated, the degree of phosphorylation in N-terminally or C-terminally truncated mutant proteins was investigated. Cell extracts were prepared from WT cells transformed with plasmids carrying the truncated FLAG-tagged *top2*^+^ genes. Mildly overproduced N-truncated Top2 mutant proteins (ΔN75 or ΔN231) produced smeary low-mobility bands that disappeared after PPase treatment as well as full-length Top2 proteins (Fig. 1*C*, *6% gel*). However, C-truncated mutant proteins (ΔC1311 or ΔC1197) abolished the phosphorylation shift of the protein bands. In addition, the short fragments ΔN1198 and ΔN1312, which correspond to the C-terminal 175 or 289 residues, produced multiple low-mobility bands (Fig. 1*C*, *12% gel*), suggesting that the Top2 C terminus 289 residues are highly phosphorylated.

We further performed Phos-tag analysis of C-terminally truncated Top2 proteins under the native promoter using a stepwise truncation series. Cells expressing ΔC1448 and ΔC1392 Top2 protein produced phosphorylation bands in the absence of PPase treatment (Fig. 1*D*). In contrast, ΔC1352 and ΔC1311 proteins showed no detectable phosphorylation. Thus, phosphorylation sites of Top2 were predicted between residues 1353 and 1392 in the C-terminal region.

Between residues 1353 and 1392, two putative CKII target residues, Ser^1363^ and Ser^1364^, have been predicted (25). These residues fit the consensus target sequence for CKII phosphorylation (S/T-*X-X*-D/E, where *X* indicates any amino acid) (36–38) (Fig. S1). Although these serine residues are not conserved among seven organisms, acidic residues are enriched around them (Fig. 1*E*). To test whether Ser^1363^ and Ser^1364^ were phosphorylated, we constructed *S. pombe* strains in which Ser^1363^ and/or Ser^1364^ were chromosomally replaced with alanine, which is unphosphorylatable. As a result of Phos-tag analysis (Fig. 1*F*), single S1363A or S1364A mutant proteins significantly reduced the smeary phosphorylated bands in the absence of PPase. In double S1363A + S1364A mutant cells, the mobility shift of the Top2 bands was hardly seen. These results indicate that both Ser^1363^ and Ser^1364^ in the *S. pombe* Top2 CTD are phosphorylated.

### Detection of Top2 phosphorylation sites by MS

To verify the Top2 phosphorylation sites identified by Phos-tag analysis, we also performed mass spectrometric analysis of the protein. Mitotic cells expressing FLAG-tagged Top2 proteins under the native promoter were collected, and Top2 proteins were immunoprecipitated by anti-FLAG antibody (Fig. S2*A*). LC-MS analysis of precipitated Top2 protein identified six putative phosphorylation sites at Ser^1310^, Ser^1363^, Ser^1416^, Thr^1417^, Ser^1431^, and Ser^1433^, all of which were located in the C-terminal region (Fig. S2, *B* and *C*). Thus, phosphorylation at Ser^1363^ was confirmed by mass spectrometric analysis but that at Ser^1364^ was not. Previous comprehensive phosphorylation analyses in *S. pombe* cells reported both Ser^1363^ and Ser^1364^ as phosphorylation sites (39–42). The reason why our analysis failed to detect Ser^1364^ phosphorylation is unclear. Further consideration of experimental conditions may be required.

### Preparation of polyclonal antibodies against two phosphopeptides containing phosphorylated Ser^1363^ or Ser^1364^

**Figure 2.**
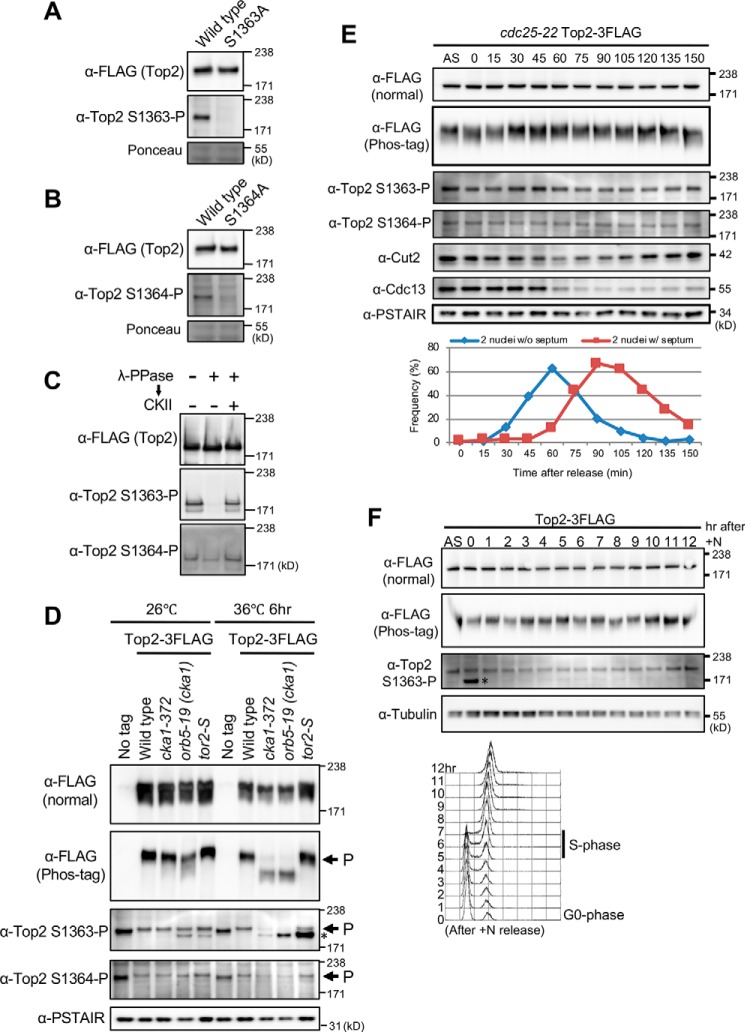
**Top2 Ser^1363^ and Ser^1364^ are phosphorylated by CKII throughout the cell cycle.** *A* and *B*, the specificity of polyclonal antibodies against phospho-Ser^1363^–containing (*A*) and phospho-Ser^1364^–containing (*B*) phosphopeptides was examined. Asynchronous extracts of the strain expressing FLAG-tagged WT Top2 protein or nonphosphorylatable alanine mutants of Top2 (S1363A or S1364A) proteins were prepared and run on SDS-PAGE. Ponceau staining was used for the loading control of extracts. *C*, Ser^1363^ and Ser^1364^ were phosphorylated by CKII *in vitro*. Immunoprecipitated Top2-FLAG proteins were dephosphorylated by λ protein phosphatase and then incubated with CKII. Anti-Top2 phospho-Ser^1363^ and -phospho-Ser^1364^ antibodies were used to detect rephosphorylation by CKII. *D*, phosphorylation of Ser^1363^ and Ser^1364^ was diminished in two distinct alleles of CKII ts mutants, *cka1-372* and *orb5/cka1-19*, at the restrictive temperature (36 °C for 6 h). Cell extracts were prepared in WT, *cka1-372*, *orb5/cka1-19* and *tor2-S* mutants expressing Top2-FLAG protein at 26 °C and 36 °C (6 h) along with the untagged strain. The *tor2-S* mutant was used as a control strain (55), which shows small cells as observed in *cka1-372* and *cka1/orb5-19* mutant cells at the restrictive temperature (Fig. S3*A*). Top2 proteins were detected with anti-FLAG antibodies in the presence (*Phos-tag*) or absence (*normal*) of 25 μm Phos-tag. Top2 Ser^1363^ and Ser^1364^ phosphorylation was detected using anti-phospho-Ser^1363^ and anti-phospho-Ser^1364^ antibodies, respectively. Anti-PSTAIR (Cdc2) antibody was used for a loading control of extracts. The *asterisk* indicates nonspecific bands that probably appear under delay or arrest of cell-cycle progression, such as under nitrogen starvation (*F*), UV irradiation (Fig. S5), and low-glucose conditions (Figs. S4*A* and S5). Because FLAG tagging partly reduces the Top2 protein level (Fig. S4*B*), both phospho-specific antibodies give a weaker signal in FLAG-tagged strains relative to untagged strains. *E*, Ser^1363^ and Ser^1364^ were phosphorylated throughout the cell cycle. Block and release of *cdc25-22* mutant cells expressing Top2–3FLAG was done for synchronous culture commencing from late G_2_ phase to mitosis. Immunoblotting was performed with antibodies against FLAG, Top2 phospho-Ser^1363^ and phospho-Ser^1364^. Cut2 (securin) and Cdc13 (mitotic cyclin) are shown as mitotic progression markers. Cell cycle progression was monitored by counting the number of binucleate cells lacking (*blue*, anaphase–telophase) and possessing septa (*red*, G_1_/S phase). *F*, *top panel*, Top2 phosphorylation was examined in nitrogen-starved, WT, G_0_-arrested cells, which were then permitted to proliferate by addition of a nitrogen source (44). Immunoblotting was performed with antibodies against FLAG, Top2 phospho-Ser^1363^, and tubulin (a loading control) as shown in *D. Bottom panel*, FACScan analysis indicating the timing of S phase (5–6 h). Top2 phosphorylation did not change during nitrogen starvation or after release.

To detect phosphorylation of the two CKII sites, antibodies were raised against two phosphopeptides containing phosphorylated Ser^1363^ or Ser^1364^ residues (phospho-Ser^1363^ or phospho-Ser^1364^) (see “Experimental procedures”). The resulting antibodies were then used for immunoblotting of FLAG-tagged Top2 in WT and alanine mutant cell extracts (S1363A or S1364A). Phosphorylated Ser^1363^ and Ser^1364^ bands were detected in WT cell extracts but were almost undetectable in the alanine mutants, demonstrating that the phosphopeptide antibodies were specific for phospho-Ser^1363^ or phospho-Ser^1364^ (Fig. 2, *A* and *B*).

We tested *in vitro* phosphorylation of Top2 by CKII. Immunoprecipitated *S. pombe* Top2 proteins were treated with λ protein phosphatase, and the resulting dephosphorylated Top2 proteins were incubated with human CKII protein complex (α and β subunits) (“Experimental procedures”). Anti-Top2 phospho-Ser^1363^ and phospho-Ser^1364^ antibodies detected the rephosphorylated Top2 protein bands in an immunoblot assay, suggesting that CKII directly phosphorylates Ser^1363^ and Ser^1364^ residues (Fig. 2*C*).

### Phosphorylation of Ser^1363^ and Ser^1364^ is diminished in CKII temperature-sensitive mutants

To determine whether phosphorylation of Top2 Ser^1363^ and Ser^1364^ depended on CKII, phospho-antibodies were used to immunoblot cell extracts obtained from two distinct ts mutants of the CKII catalytic subunit Cka1, *cka1–372* (newly identified in this work) and *cka1/orb5-19* (43) (Fig. S3*A*). The mutation site of *cka1–372* is identified as H152Y, which is located close to the kinase active site (Fig. S3*B*). At 26 °C, phosphorylated Ser^1363^ and Ser^1364^ bands were detected by specific phospho-antibodies in all tested cell extracts (Fig. 2*D*). In Phos-tag analysis, there are no significant differences in electrophoretic mobility of Top2–3FLAG proteins among these extracts. At 36 °C, however, phospho-Ser^1363^ and phospho-Ser^1364^ were diminished in both *cka1–372* and *cka1/orb5-19* mutant cells. Consistently, Top2 proteins showed faster mobility in Phos-tag gels in these mutants, strongly suggesting that CKII is responsible for phosphorylation of Top2 at residues Ser^1363^ and Ser^1364^.

### Top2 Ser^1363^ and Ser^1364^ are constitutively phosphorylated throughout the cell cycle

We then performed a block–release experiment using the *cdc25-22* ts mutant to examine whether phosphorylation of Ser^1363^ and Ser^1364^ changes in any cell cycle stage. *cdc25-22* cells expressing Top2–3FLAG were blocked in late G_2_ and released synchronously into mitosis by a shift in temperature from 36 °C to 26 °C. Bands of phospho-Ser^1363^ and phospho-Ser^1364^ were almost constant throughout the cell cycle, although phospho-Ser^1363^ and overall phosphorylation of Top2–3FLAG (Phos-tag gel) were slightly more intense at 30 and 45 min, which correspond to early mitosis (Fig. 2*E*).

We also examined Top2 phosphorylation in nitrogen-starved, WT, G_0_-arrested cells, which were then permitted to proliferate by addition of a nitrogen source (44). The level of phosphorylation at Top2 Ser^1363^ in G_0_ was comparable with that in asynchronous culture and not significantly changed in proliferative cells (Fig. 2*F*). Therefore, these results indicate that CKII-dependent phosphorylation at Ser^1363^ and Ser^1364^ is maintained throughout the cell cycle.

### Unphosphorylatable Top2-2A mutant protein maintains DNA decatenation activity but partly decreases ATPase activity

**Figure 3.**
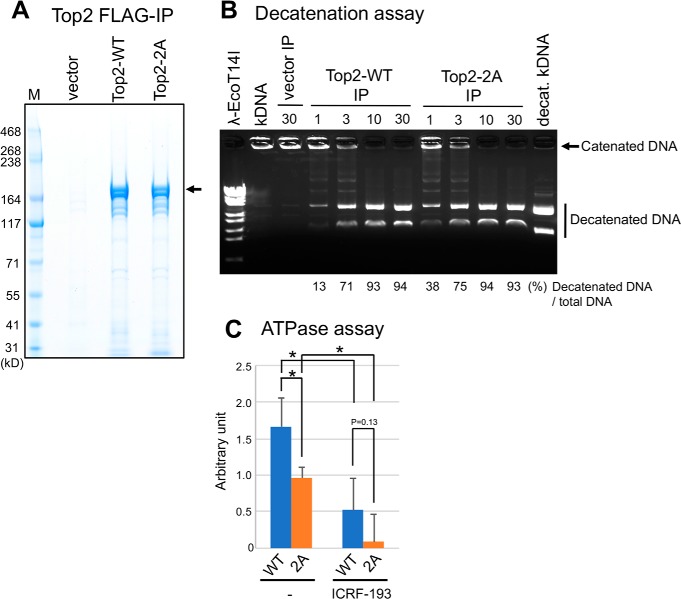
**Top2–2A (S1363A,S1364A) mutant protein maintains decatenation activity but has reduced ATPase activity.** *A*, SDS-PAGE patterns of WT Top2 and alanine substitution mutant Top2-2A (S1363A and S1364A). FLAG-tagged Top2-WT and -2A proteins were overproduced under the inducible nmt promoter (plasmid Rep41) in WT *S. pombe* cells and immunoprecipitated using anti-FLAG antibody. A strain containing only the vector was used as a control. Immunoprecipitated FLAG-tagged Top2 proteins are indicated by *arrows*. The position of the protein marker bands (*M*) is indicated. *B*, Top2 decatenation assay. kDNA (153 ng) was incubated with immunoprecipitated Top2 fractions in ATP-containing reaction buffer (see “Experimental procedures”) for 1–30 min at 37 °C. An immunoprecipitated fraction from cell extracts containing only the empty vector was used for a mock reaction (*vector IP*). The reaction was terminated using stop buffer and loaded onto a 1% agarose gel followed by ethidium bromide staining. Only the catenated kDNA (*kDNA*) and decatenated kDNA (*decat. kDNA*) were loaded as controls, along with λDNA digested by EcoT14I (λ*-EcoT14I*). Positions of catenated and decatenated kDNA are indicated by an *arrow* and a *vertical line*, respectively. The ratio of decatenated DNA to total DNA (catenated + decatenated) was quantified. Phosphorylation of Ser^1363^ and Ser^1364^ does not affect Top2 decatenation activity. *C*, Top2 ATPase assay. Immunoprecipitated Top2-WT or -2A mutant proteins were incubated with ATP and kDNA in the presence or absence of the 5 μm anti-cancer topo II inhibitor ICRF-193 for 30 min at 30 °C. Free phosphate produced by ATP hydrolysis was measured by malachite green colorimetric reagent (see “Experimental procedures”). *Error bars* represent the standard deviation for each experiment performed in triplicate. *p* values for comparison among four conditions were calculated using one-way analysis of variance with Holm multi-comparison correction. *, *p* < 0.05.

*S. pombe* Top2 protein dephosphorylated by potato acid phosphatase is still enzymatically active (25). To address the question of whether the enzymatic activity of Top2 is affected by phosphorylation of Ser^1363^ and Ser^1364^, we analyzed DNA decatenation activity using Top2 WT and unphosphorylatable 2A (S1363A S1364A) mutant proteins. FLAG-tagged Top2-WT and -2A proteins were overproduced under the inducible nmt promoter in WT cells and immunoprecipitated using anti-FLAG antibody (Fig. 3*A*). Immunoprecipitated Top2 proteins were incubated with kinetoplast DNA (kDNA) in the presence of ATP for 1–30 min at 37 °C (see “Experimental procedures”). The results indicate that both Top2-WT and -2A proteins decatenated kinetoplast DNA within 10 min (Fig. 3*B*). Thus, consistent with previous work, CKII-dependent phosphorylation at Ser^1363^ and Ser^1364^ presumably did not affect the decatenation activity of Top2.

Next we assayed the ATPase activity of Top2 WT and 2A mutant proteins. Immunoprecipitated Top2 proteins were incubated with ATP in the presence of kDNA for 30 min, and resulting free phosphate was measured by malachite green colorimetric assay (see “Experimental procedures”). The ATPase activity of unphosphorylatable Top2 2A mutant proteins partly decreased to ∼60% compared with WT proteins (Fig. 3*C*). In the presence of an anticancer drug, ICRF-193, which inhibits the ATPase activity of topo II protein (15, 16), both WT and 2A mutant proteins lost much of their ability to hydrolyze ATP. This result suggests that CKII-mediated Top2 phosphorylation at Ser^1363^ and Ser^1364^ facilitates ATP hydrolysis.

### More severe defects in chromosome segregation occur in cka1–372 mutant cells treated with a topo II inhibitor, ICRF-193

**Figure 4.**
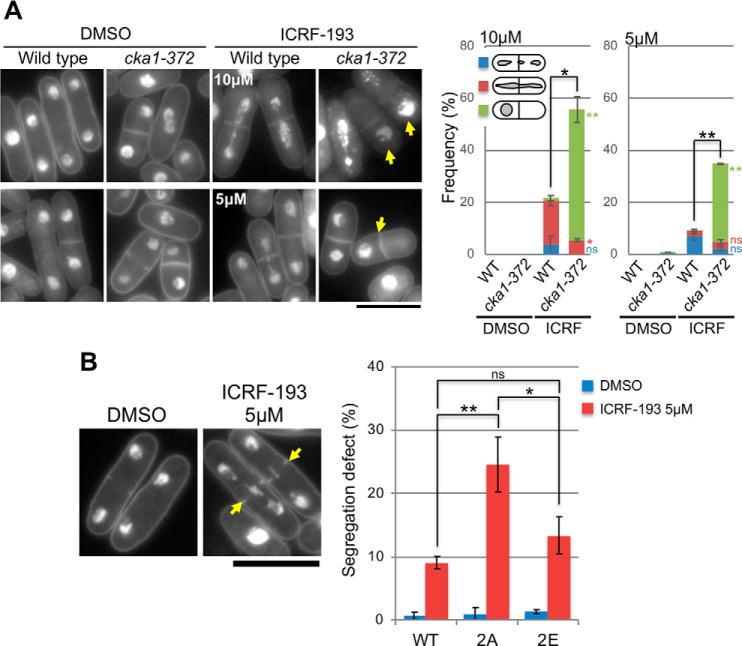
**Defective chromosome segregation induced by an anti-cancer catalytic topo II inhibitor, ICRF-193, is exacerbated in *cka1-372* and *top2-2A* (S1363A,S1364A) mutant cells.** *A*, CKII ts mutant *cka1-372* cells showed more severe defects in mitotic chromosome segregation than WT cells in the presence of ICRF-193. *Left panel*, DAPI-stained micrographs of WT and *cka1-372* mutant cells were obtained at the restrictive temperature (36 °C) for 3 h in the presence of DMSO or ICRF-193 (5 and 10 μm). A displaced nuclear phenotype was frequently observed in ICRF-193–treated *cka1* mutant cells (*arrows*). *Right panel*, frequencies of defective phenotypes categorized as lagging-like (*blue*), streaked chromosomes (*red*), and displaced nucleus (*green*). More than 200 anaphase cells were counted for each sample. *Error bars* represent the standard deviation for each defective phenotypes. *p* values for comparison between the drug-treated WT and *cka1* mutant were calculated using a Student's *t* test. *, *p* < 0.05; **, *p* < 0.01 (*black*, total frequency of abnormal phenotypes; *color*, each phenotype). *B*, defects in chromosome segregation increased significantly in unphosphorylatable *top2-2A* mutants compared with WT and phosphomimetic *top2–2E* mutant cells in the presence of ICRF-193. Cells were asynchronously cultured at 26 °C for 2 h in the presence of DMSO or 5 μm ICRF-193. *Left panel*, representative micrographs of DMSO- and 5 μm ICRF-193–treated *top2-2A* cells. Chromatin DNA was stained with DAPI. Abnormally streaked chromosomes in anaphase are indicated (*arrows*). *Scale bar* = 10 μm. *Right panel*, frequencies of anaphase cells with abnormally streaked chromosomes. More than 200 anaphase cells were counted for each sample. *Error bars* represent the standard deviation for each experiment performed in biological triplicates. Significant differences among the three strains were examined using one-way analysis of variance with Bonferroni multi-comparison correction. *, *p* < 0.05; **, *p* < 0.01; *n.s.*, not significant.

To investigate the functional relationship between CKII and Top2 in mitosis, we analyzed chromosome segregation in *cka1–372* mutant cells in the presence of ICRF-193. WT and *cka1–372* mutant cells were cultivated at 26 °C and then shifted to 36 °C for 3 h in the presence or absence of 5 or 10 μm ICRF-193. In the absence of the drug (mock treatment with DMSO), neither WT nor *cka1* mutant cells produced abnormal segregation of mitotic chromosomes (Fig. 4*A*). On the other hand, the *cka1* mutant failed to segregate chromosomes to a greater extent than WT cells in the presence of 5 or 10 μm ICRF-193. The drug-treated *cka1* mutant showed a “displaced nucleus” in one daughter cell, which is a more severe segregation defect than partial mis-segregation, such as lagging or streaked chromosomes, as observed in drug-treated WT cells. These observations indicated that inhibition of Top2 activity and loss of CKII function additively interfere with mitotic chromosome segregation.

### ICRF-193–induced chromosome mis-segregation is exacerbated in top2-2A mutant cells

To determine the contribution of phospho-Ser^1363^ and phospho-Ser^1364^ to chromosome segregation, we observed the mitotic phenotype of Top2 WT, 2A unphosphorylatable, and phosphomimetic 2E mutant cells in the presence or absence of ICRF-193. DMSO treatment did not cause segregation defects in any tested strains (Fig. 4*B*). In ICRF-193–treated WT cells, 9% of anaphase cells showed abnormally streaked chromosomes; however, the frequency of defective segregation was significantly increased in *top2-2A* mutant cells, in which 25% of anaphase cells produced abnormally streaked chromosomes. Segregation defects in *top2-2E* mutant cells were observed to a lesser extent than in 2A mutant cells. Thus, phosphorylation at Ser^1363^ and Ser^1364^ apparently increases the fidelity of chromosome segregation when Top2 function is impaired by ICRF-193.

## Discussion

So far as we are aware, our results provide the first evidence that CKII-mediated phosphorylation of the topo II CTD suppresses sensitivity to topo II catalytic inhibitors. Topo II phosphorylation confers resistance to DNA double-strand break–inducing topo II poisons on mammalian cells (45, 46). Actually, the phosphorylation level of topo II protein is increased in topo II poison–resistant human cancer cells (47). Thus, when either type of anti-topo II drug is applied, inhibition of topo II phosphorylation may additively interfere with mitotic chromosome segregation.

**Figure 5.**
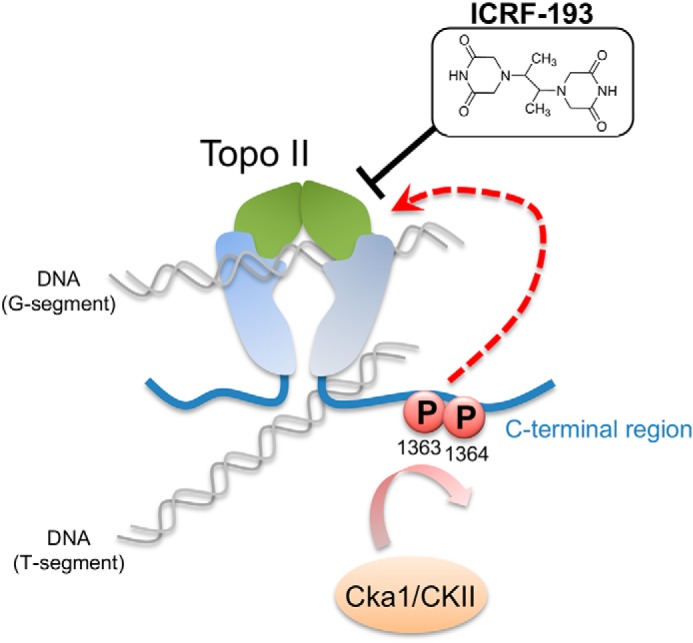
**Cartoon of the possible action of phosphorylated topo II residues Ser^1363^ and Ser^1364^ on the ATP-binding and hydrolysis domain targeted by ICRF-193.** An ATP-binding hydrolytic domain (*green*), a DNA-binding cleavage domain (*light blue*), and a C-terminal region (*blue*) are illustrated with gate (*G*) and transport (*T*) segments of dsDNAs. The *T-shaped line* represents the inhibition of the ATP-binding hydrolytic domain by ICRF-193 (a structural formula is shown). The *dashed arrow* represents the possible action of the topo II residues Ser^1363^ and Ser^1364^ phosphorylated by CKII. See text for more details.

How does topo II phosphorylation diminish the inhibitory effect of ICRF-193 on chromosome segregation? ICRF-193 traps the topo II–DNA complex in a closed, clamped configuration by inhibiting its ATPase activity (15, 16). Contrarily, ATP hydrolysis is stimulated 2.7-fold after topo II phosphorylation by CKII in *Drosophila* cells (29). Therefore, ICRF-193 and CKII-mediated topo II phosphorylation have an antagonistic effect on the ATPase activity of topo II. Topo II hydrolyzes ATP at the end of the catalytic cycle after dsDNA passage and religation reactions and is a prerequisite for topo II turnover on DNA (29). It is thus suggested that ICRF-193 interferes with the release of topo II protein from chromosome DNA; however, CKII promotes topo II translocation along the DNA through phosphorylation. In this study, we show that Top2 unphosphorylatable 2A (S1363A, S1364A) mutations partly decrease ATPase activity (Fig. 3*C*). The phosphorylated topo II residues Ser^1363^ and Ser^1364^ may directly modulate the ATP-binding catalytic domain, which is targeted by ICRF-193, or indirectly associate with the domain through DNA binding (Fig. 5). In the presence of ICRF-193, we could not detect a statistically significant difference in ATPase activity between Top2-WT and -2A proteins, implying that additional phosphorylation sites may be involved in modulation of the activity. Further study is required to understand the molecular action of the phosphorylated topo II CTD on its catalytic ATPase domain.

It has been reported that topo II CTD is mitotically SUMOylated and that this modification is required for Topo II accumulation at centromeres in *Xenopus* egg extracts (32, 48–50). Phosphorylated topo II protein was also enriched at centromeric DNA in human cells (51, 52). This evidence supports the idea that posttranslational modification of topo II CTD modulates association and dissociation of topo II protein with chromosome DNA.

We demonstrated that CKII-dependent phosphorylation at the Top2 residues Ser^1363^ and Ser^1364^ is maintained throughout the entire cell cycle. This result is consistent with the constitutive activation of CKII in various organisms (53, 54). CKII is a ubiquitous, essential kinase for a wide variety of cellular functions, including transcription, heat shock response, target of rapamycin complex–mediated nutrient signaling, and maintenance of cell morphology, indicating its pivotal role in cell proliferation (38, 43, 55–59). Therefore, it is assumed that CKII persistently relieves the topological constraints upon DNA through the phosphorylation of Top2 Ser^1363^ and Ser^1364^ at any cell cycle stage. To deal with simultaneous and multiple occurrences of topological stress along chromosomal DNA, constitutive phosphorylation of the Top2 CTD by CKII is probably essential for the continuous action of Top2 molecules.

The phosphorylation status of Top2 Ser^1363^ and Ser^1364^ did not change under any tested stressful conditions, including UV exposure, heat shock, osmotic stress, low glucose, and nitrogen starvation (Fig. S5). In addition, *top2-2A* mutant strains did not show any sensitivities to DNA damage–inducing agents or nutritional limitation as far as we could determine, suggesting that phosphorylation at Top2 Ser^1363^ and Ser^1364^ is not involved in these stress responses. An alternative possibility is that the constitutively phosphorylated Top2 CTD is targeted by specific factor(s) and loaded onto chromosomal DNA under stressful physiological conditions.

CKII protein levels are consistently higher in tumors than in normal tissues, and overexpression of CKII has been reported to cause neoplastic growth in animal cells (60). These facts have increased interest in CKII as a target of anticancer chemotherapy (61–63). Indeed, potent inhibitors against CKII have been developed and applied (64, 65). We assume that higher amounts of anti-topo II drugs may be required to inhibit mitotic progression in which hyperactivated CKII accelerates cell proliferation. Thus, our results suggest possible benefits of applying CKII inhibitors in combination with topo II catalytic inhibitors for effective prevention of mitotic chromosome segregation in tumor cells.

## Experimental procedures

### Strains, plasmids, and media

The *S. pombe* haploid WT strain 972 *h*^−^ and its derivative mutant strains, including the ts *orb5-19* (43), *tor2-S* (*L2048S*) (55), *cdc25-22* (66) and the cs *nda3-KM311* (67), were used. The ts *cka1–372* mutant was newly isolated from a collection of 1015 ts strains (68). A strain with chromosomally integrated 3FLAG-tagged Top2 has been described previously (35). For Top2 alanine or glutamate mutants, site-directed PCR-based mutagenesis was used. Briefly, complementary pairs of oligonucleotide DNAs with mutations were used as PCR primers, followed by two rounds of PCR. The mutated *top2* gene was cloned and chromosomally integrated with the FLAG tag, the *adh1*^+^ 3′ UTR, and the kanamycin-resistant gene *kan*^R+^ into the endogenous *top2* locus of the WT strain 972 *h*^−^. Correct integration was confirmed by PCR and digestion of the PCR products with restriction enzymes. To construct the 3FLAG-tagged Top2 C-terminal deletion mutants, *top2*^+^ gene fragments were amplified by PCR and chromosomally integrated under the native promoter in the same manner. pRep81 plasmids expressing FLAG-tagged N- and/or C-terminal deletion mutant proteins were constructed by PCR amplification. ICRF-193 (Zenyaku Kogyo, Tokyo, Japan) was dissolved in DMSO and added to culture media to a final concentration of 5 or 10 μm (20).

### Synchronous culture

For the block-and-release experiment using the *cdc25-22* mutant (66), cells were grown in YPD at 26 °C (to 3 × 10^6^ cells/ml) and then shifted to 36 °C for 4.25 h to block cells in late G_2_ phase. Cells were then released to 26 °C, and aliquots were taken every 15 min for immunoblotting and determination of the septation index. Mitotically arrested cells using the *nda3-KM311* cold-sensitive mutant were prepared as described previously (67). For nitrogen starvation and release, cells were transferred to nitrogen-deficient Edinburgh Minimal Medium 2 (EMM2-N) medium at 26 °C for 24 h and then replenished by addition of 0.5% NH_4_Cl (44). FACScan analysis was carried out as described previously (20).

### Immunochemistry and phosphate-affinity SDS-PAGE

Protein extracts were prepared by cell breakage using glass beads in extraction buffer (25 mm Tris-HCl (pH 7.5), 0.1% NP-40, 10% glycerol, and 1 mm DTT) supplemented with protease inhibitor mixture (Sigma). Extracts were boiled with lithium dodecyl sulfate (LDS) sample buffer (Invitrogen) and loaded onto 6% or 12% polyacrylamide gels. Phosphate-affinity SDS-PAGE was carried out using Phos-tag® acrylamide (NARD Institute) following the manufacturer's instructions. λ PPase (New England Biolabs) was used for phosphatase treatment. PPase-treated or untreated cell extracts were run on polyacrylamide gels containing Phos-tag acrylamide and MnCl_2_. To increase transfer efficiency, manganese ions were eliminated from the gel by soaking it in transfer buffer containing 1 mm EDTA. Immunoblotting was performed using the following antibodies: anti-FLAG M2 (Sigma), anti-PSTAIR (a gift from Dr. Y. Nagahama, National Institute for Basic Biology, Okazaki, Japan), anti-Cut2 (69), and anti-Cdc13 (70). Top2 phospho-antibodies were obtained by immunizing rabbits with synthetic phosphopeptides (Sigma): (1351-RKTNKPVATTIF[S-phos]SDDEDD-1369) for anti-Top2 phospho-Ser^1363^ and (1351-RKTNKPVATTIFS[S-phos]DDEDD-1369) for anti-Top2 phospho-Ser^1364^. Intensity of protein bands was measured using the image analysis software ImageJ (National Institutes of Health). Mass spectrometric analysis for identification of Top2 phosphorylation sites was performed as described previously (10).

### In vitro phosphorylation of Top2 protein

To prepare Top2-WT protein, pRep41 plasmid expressing the protein under the inducible nmt promoter was transformed in the *S. pombe* WT strain. The resulting strain was cultured at 26 °C for 24 h in the absence of thiamine to induce FLAG-tagged Top2 proteins, and then 1 × 10^9^ cells were collected. Frozen cells were disrupted and immunoprecipitated using anti-FLAG M2–agarose beads (Sigma) as described previously (10). For the *in vitro* phosphorylation assay, immunoprecipitated Top2 proteins (∼20 ng) were treated with 400 units of λ protein phosphatase (New England Biolabs, P0753) for 30 min at 30 °C. Dephosphorylated Top2 proteins were incubated with 500 units of casein kinase II protein complex (α and β subunits) derived from human cells (New England Biolabs, P6010) in the presence of 200 μm ATP for 30 min at 30 °C. Rephosphorylated Top2 proteins were immunoblotted by anti-Top2 phospho-Ser^1363^ and phospho-Ser^1364^ antibodies.

### Fluorescence microscopy

DAPI staining was carried out as described previously (71). All-in-one microscopes (BZ9000 and BZ-X700, Keyence), were used to observe glutaraldehyde-fixed cells.

### Top2 decatenation assay

pRep41 plasmids expressing FLAG-tagged Top2-WT or -2A proteins under the inducible nmt promoter were constructed and transformed in the *S. pombe* WT strain. The resulting strains were cultured at 26 °C for 24 h in the absence of thiamine, and then Top2-FLAG proteins were immunoprecipitated using anti-FLAG M2–agarose beads (Sigma) as described previously (10). Top2 decatenation assays were performed using the Topoisomerase II Assay Kit (TopoGEN, Inc., TG1001-1). Kinetoplast DNA (153 ng) was incubated with the Top2 IP fraction (∼5 ng) at 37 °C for 1–30 min in reaction buffer (50 mm Tris-HCl (pH 8.0), 150 mm NaCl, 10 mm MgCl_2_, 0.5 mm DTT, 30 μg/ml BSA, and 2 mm ATP). The reaction was terminated with stop buffer (1% sarkosyl, 0.025% bromophenol blue, and 5% glycerol). Samples were run on a 1% agarose gel in 1× Tris borate-EDTA at 135 V for 25 min and stained with ethidium bromide. Intensity of DNA bands was measured using ImageJ (National Institutes of Health).

### Top2 ATPase assay

*S. pombe* Top2 proteins were immunoprecipitated as described above and subjected to a malachite green assay using an ATPase/GTPase activity assay kit (Sigma, MAK113). Kinetoplast DNA (270 ng) was incubated with the Top2 IP fraction (0.1 μg) in assay buffer containing 40 mm Tris, 80 mm NaCl, 8 mm MgAc_2_, and 1 mm EDTA (pH 7.5). Assays were performed in the presence or absence of 5 μm ICRF-193 at 30 °C for 30 min in triplicate. The resulting colorimetric product was measured at 600 nm with a spectrophotometric plate reader (ARVO X3, PerkinElmer Inc.). The immunoprecipitated fraction from a strain expressing an empty vector was also assayed and used as a background control. Background readings could then be subtracted from sample readings.

## Author contributions

N. N. and M. Y. conceptualization; N. N. data curation; N. N. formal analysis; N. N. and M. Y. validation; N. N. investigation; N. N. visualization; N. N., O. A., and M. E. methodology; N. N. writing-original draft; N. N. and M. Y. writing-review and editing; M. E. software; M. Y. supervision.

## Supplementary Material

Supporting Information

## Footnotes

3 The abbreviations used are:

topo II: DNA topoisomerase II
CTD: C-terminal domain
PPase: protein phosphatase
cDNA: complementary DNA
kDNA: kinetoplast DNA
ts: temperature-sensitive
cs: cold-sensitive
DAPI: 4′,6-diamidino-2-phenylindole
IP: immunoprecipitation.
