# Supplementary material for: Casein kinase II–dependent phosphorylation of DNA topoisomerase II suppresses the effect of a catalytic topo II inhibitor, ICRF-193, in fission yeast
Source: J Biol Chem. 2019 Jan 11;294(10):3772–82. doi: 10.1074/jbc.RA118.004955 (PMC6416453; doi:10.1074/jbc.RA118.004955)
Supplement: Supporting Information [file supp_294_10_3772__index.html]

Casein kinase II-dependent phosphorylation of DNA topoisomerase II suppresses the effect of a catalytic topo II inhibitor, ICRF-193, in fission yeast — Effect of topo II phosphorylation on ICRF-193 treatment — Casein kinase II–dependent phosphorylation of DNA topoisomerase II suppresses the effect of a catalytic topo II inhibitor, ICRF-193, in fission yeast — Effect of topo II phosphorylation on ICRF-193 treatment — Supporting Information 

# Casein kinase II–dependent phosphorylation of DNA topoisomerase II suppresses the effect of a catalytic topo II inhibitor, ICRF-193, in fission yeast

## Supporting Information

- Supporting Information (to be published online) - Supplemental Figures 1-5 and table 1
