# Supplementary material for: Casein kinase II–dependent phosphorylation of DNA topoisomerase II suppresses the effect of a catalytic topo II inhibitor, ICRF-193, in fission yeast
Source: J Biol Chem. 2019 Jan 11;294(10):3772–82. doi: 10.1074/jbc.RA118.004955 (PMC6416453; doi:10.1074/jbc.RA118.004955)
Supplement: Supporting Information [file supp_RA118.004955_139536_3_supp_265806_pl4hbk.pdf]

Supporting information

Casein kinase II-dependent phosphorylation of DNA topoisomerase II suppresses the effect of a catalytic topo II inhibitor, ICRF-193, in fission yeast

Norihiko Nakazawa[1*], Orie Arakawa[1], Masahiro Ebe[1], and Mitsuhiro Yanagida[1*]

[1]G0 Cell Unit, Okinawa Institute of Science and Technology Graduate University, Onna-son, Okinawa 904-0495, Japan

*Corresponding authors: nakazawa@oist.jp and myanagid@gmail.com

Supplemental Figure S1. Amino acid sequence of the *S. pombe* Top2 C-terminal region

A schematic representation of *S. pombe* Top2 polypeptide and the amino acid sequence of the Top2 C-terminal region (1198-1485 residues) are shown. Acidic and basic residues are indicated in red and blue, respectively, with putative NLS sequences and identified phosphorylation sites (S1363 and S1364). Top2 C-terminal 40 amino acids between residues 1353 and 1392, in which phosphorylation sites are predicted, are indicated.

Supplemental Figure S2. Detection of Top2 phosphorylation sites by mass spectrometry

(A) Experimental procedure for identification of Top2 phosphorylation by LC-MS analysis. **(B)** S1310, S1363, S1416, T1417, S1431, and S1433 were identified as putative phosphorylation sites in the Top2 C-terminal region (blue box). The coverage of detected peptides of Top2 was 73 % (red characters). **(C)** List of detected peptides including phosphorylated serine or threonine residues. Ion score values (obtained from Mascot software, Matrix Science, UK) are indicated.

Supplemental Figure S3. Cell phenotypes of temperature sensitive *cka1-372*, *orb5 (cka1)-19*, and *tor2-S* mutants at the restrictive temperature (36°C) for 6 hr

(A) DAPI-stained micrographs of wild-type, *cka1-372*, *orb5 (cka1)-19*, and *tor2-S* mutant cells are shown. Scale bar, 10µm. **(B)** The amino acid sequence of *S. pombe* Cka1 protein, with orthologs of alpha catalytic subunit of CKII in human (HsCSNK2A1 and HsCSNK2A2) and budding yeast (ScCKA1 and CKA2). *S. pombe* possesses Cka1 as the sole alpha catalytic subunit of CKII. Mutation site of the *cka1-372* ts mutant and the active site of serine/threonine-protein kinase are indicated in red and blue boxes, respectively.

Supplemental Figure S4. Validation of non-specific bands recognized by anti-Top2

S1363-P, and comparison of protein level between untagged and FLAG-tagged Top2 proteins

(A) Under the 0.08% glucose condition, anti-S1363-P antibody recognized the additional band around 171 kD in western blots (red asterisks). Top2-S1363A mutant protein and deletion of Top2 C-terminus (ΔC1352, which lacked the S1363 residue) showed the additional band at the same position, as well as full length Top2 protein, suggesting that the band is not derived from Top2 protein. Immunoblotting was performed as shown in Figure 2. **(B)** Immunoblotting of Top2 protein using an antibody against the Top2 protein (33). The anti-Top2 antibody gave weak signal in FLAG-tagged strain relative to the untagged strain, indicating that the FLAG-tagging partly reduces the Top2 protein level. FLAG-tagged Top2 strain is indistinguishable from untagged strain with regards to their cell growth, drug sensitivity, and mitotic phenotype, as far as we tested.

Supplemental Figure S5. Top2 phosphorylation was maintained under stressful conditions

Top2 phosphorylation was examined under UV, heat shock, osmotic stress, and glucose starvation treatments as indicated. Immunoblotting was performed with antibodies against FLAG, Top2 S1363-P, Top2 S1364-P, along with Ponceau staining (a loading control). The asterisk indicates non-specific bands, which probably appear under delay or arrest of cell-cycle progression, as described in the legend of Figure 2D.

Supplemental Table S1. Individual data points of Top2 ATPase assay in Figure 3C

Supplemental Figure S1

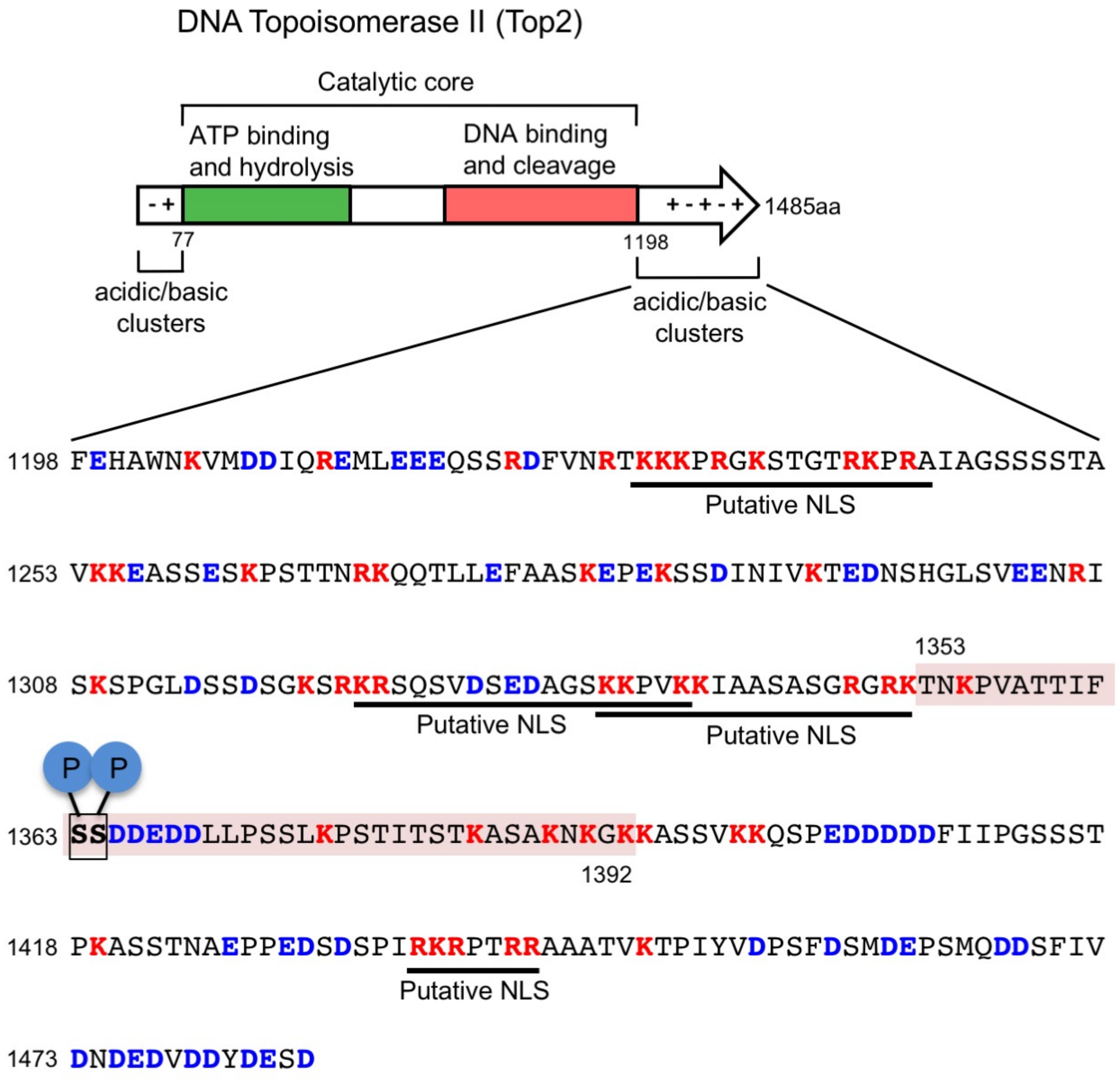

Supplemental Figure S2

A

Expression of Top2-3FLAG protein under the native promoter

↓

Immunoprecipitation of Top2-3FLAG by anti-FLAG antibody

↓

Elution of precipitated Top2-3FLAG by FLAG peptide

↓

Detection of phosphorylated residues by LC-MS analysis

B

Top2 protein

Detected peptide (coverage 73%)

☐ Detected phosphorylation sites

```
   1 MSIDADFSDY EDEASGDENV LPNTTTKRKA STTSSKSRAK KASTPDLRQT
  51 SLTSMTASEQ IPLVTNNGNG NSNVSTQYQR LTPREHVLRR PDTYIGSIEP
 101 TTSEMWVFDS EKNKLDYKAV TYVPGLYKIF DEIIVNAADN KVRDPNMNTL
 151 KVTLDPEANV ISIYNNGKGI PIEIHDKEKI YIPELIFGNL LTSSNYDDNQ
 201 KKVTGGRNGY GAKLCNIFST EFVVETADKE RMKKYKQTWY DNMSRKSEPV
 251 ITSLKKPDEY TKITFKPDLA KFGMDKIDDD MVSIIKRRIY DMAGTVRETK
 301 VYLNNERISI SGFKKYVEMY LASDTKPDEE PPRVIYEHVN DRWDVAFAVS
 351 DGQFKQVSFV NNISTIRGGT HVNYVANKIV DAIDEVVKKE NKKAPVKAFQ
 401 IKNYVQVFVN CQIENPSFDS QTKETLTTKV SAFGSQCTLS DKFLKAIKKS
 451 SVVEEVLKFA TAKADQQLSK GDGGLRSRIT GLTKLEDANK AGTKESHKCV
 501 LILTEGDSAK SLAVSGLSVV GRDYYGVFPL RGKLLNVREA SHSQILNNKE
 551 IQAIKKIMGF THKKTYTDVK GLRYGHLMIM TDQDHDGSHI KGLIINYLES
 601 SYPSLLQIPG FLIQFITPII KCTRGNQVQA FYTLPEYEYW KEANNNGRGW
 651 KIKYYKGLGT SDHDDMKSYF SDLDRHMKYF HAMQEKDAEL IEMAFAKKKA
 701 DVRKEWLRTY RPGIYMDYTQ PQIPIDDFIN RELIQFSMAD NIRSIPSVVD
 751 GLKPGQRKVV YYCFKRNLVH ETKVSRLAGY VASETAYHHG EVSMEQTIVN
 801 LAQNFVGSNN INLLMPNGQF GTRSEGGKNA SASRYLNTAL SPLARVLFNS
 851 NDDQLLNYQN DEGQWIEPEY YVPILPMVLV NGAEGIGTGW STFIPNYNPK
 901 DITANLRHML NGEPLEIMTP WYRGFRGSIT KVAPDRYKIS GIINQIGENK
 951 VEITELPIRF WTQDMKEYLE AGLVGTEKIR KFIVDYESHH GEGNVHFNVT
1001 LTEAGMKEAL NESLEVKFKL SRTQATSNMI AFDASGRIKK YDSVEDILTE
1051 FYEVRLRTYQ RRKEHMVNEL EKRFDRFSNQ ARFIHMIIEG ELVVSKKKKK
1101 DLIVELKEKK FQPISKPKKG HLVDLEVENA LAEEEQSGDV SQDEDSDAYN
1151 YLLSMPLWSL TYERYVELLK KKDEVMAELD ALIKKTPKEL WLHDLDAFEH
1201 AWNKVMDDIQ REMLEEEQSS RDFVNRTKKK PRGKSTGTRK PRAIAGSSSS
1251 TAVKKEASSE SKPSTTNRKQ QTLLEFAASK EPEKSSDINI VKTEDNSHGL
1301 SVEENRISKS PGLDSSDSGK SRKRSQSVDS EDAGSKKPVK KIAASASGRG
1351 RKTNKPVATT IFSSDDEDDL LPSSLKPSTI TSTKASAKNK GKKASSVKKQ
1401 SPEDDDDDFI IPGSSSTPKA SSTNAEPPED SDSPIRKRPT RRAAATVKTP
1451 IYVDPSFDSM DEPSMQDDSF IVDNDEDVDD YDESD
```

C

Location	Detected peptide (phospho sites)	Ion score	Phospho sites
1307 - 1320	R.ISKSPGLDSSDSGK.S	43	S1310
1353 - 1384	K.TNKPVATTIFSSDDEDDLLPSSLKPSTITSTK.A	25	S1363
1399 - 1419	K.KQSPEDDDDDFIIPGSSSTPK.A	23	T1417
1400 - 1419	K.QSPEDDDDDFIIPGSSSTPK.A	53	S1416
1400 - 1419	K.QSPEDDDDDFIIPGSSSTPK.A	29	T1417
1420 - 1437	K.ASSTNAEPPEDSDSPIRK.R	36	S1433
1420 - 1436	K.ASSTNAEPPEDSDSPIR.K	46	S1433
1420 - 1436	K.ASSTNAEPPEDSDSPIR.K	21	S1431

Supplemental Figure S3

A

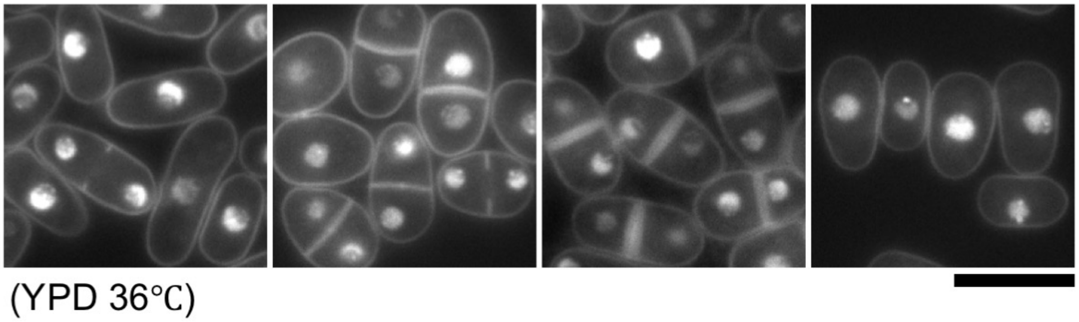

B

```
HsCSNK2A1     1 ----------MSGPVPSRARVYTDVNTHRPREYWDYES-HVVEWGNQDDYQLVRKLGRGK
HsCSNK2A2     1 ---------MPGPAAGSRARVYAEVNSLRSREYWDYEA-HVPSWGNQDDYQLVRKLGRGK
SpCka1        1 ------MNQTEAAPVVSVSRVYAHVNEEMPREYWDYEN-MQEVFGYQDNYEIIRKVGRGK
ScCKA2        1 MPLPPSTLNQKSNRVYSVARVYKNACEERPQEYWDYEQGVTIDWGKISNYEIINKIGRGK
ScCKA1        1 ----------MKCRVWSEARVYTNINKQRTEEYWDYENTVIDWSTNTKDYEIENKVGRGK

HsCSNK2A1    50 YSEVFEAINITNNEKVVVKILKPVKKKKIKREIKILENLR--------------------
HsCSNK2A2    51 YSEVFEAINITNNERVVVKILKPVKKKKIKREVKILENLR--------------------
SpCka1       54 YSEVFEGLNVLNNSKCIIKVLKPVKYKKIKREIKILQNLA--------------------
ScCKA2       61 YSEVFSGRCIVNNQKCVIKVLKPVKMKKIYRELKILTNLT--------------------
ScCKA1       51 YSEVFQGVKLDSKVKIVIKMLKPVKKKKIKREIKILTDLSNEKVPPTTLPFQKDQYYTNQ

HsCSNK2A1    90 ------------------GGPNIITLADIVKDPVSRTPALVFEHVNNTDFKQLYQTLTDY
HsCSNK2A2    91 ------------------GGTNIIKLIDTVKDPVSKTPALVFEYINNTDFKQLYQILTDF
SpCka1       94 ------------------GGPNIISLLDIVRDPESKTPSLIFEFVDNIDFRTLYPTLSDY
ScCKA2      101 ------------------GGPNVVGLYDIVQDADSKIPALIFEEIKNVDFRTLYPTFKLP
ScCKA1      111 KEDVLKFIRPYIFDQPHNGHANIIHLFDIIKDPISKTPALVFEYVDNVDFRILYPKLTDL

                  cka1-372 (H152Y) Serine/threonine-protein kinase, active site
HsCSNK2A1   132 DIRFYMYEILKALDYCHSMGIMHRDVKPHNVMIDHEHRKLRLIDWGLAEFYHPGQEYNVR
HsCSNK2A2   133 DIRFYMYELLKALDYCHSKGIMHRDVKPHNVMIDHQQKKLRLIDWGLAEFYHPAQEYNVR
SpCka1      136 DIRYYSYELLKALDFCHSRGIMHRDVKPHNVMIDHKKRKLRLIDWGLAEFYHAGMEYNVR
ScCKA2      143 DIQYYFTQLLIALDYCHSMGIMHRDVKPQNVMIDPTERKLRLIDWGLAEFYHPGVDYNVR
ScCKA1      171 EIRFYMFELLKALDYCHSMGIMHRDVKPHNVMIDHKNKKLRLIDWGLAEFYHVNMEYNVR

HsCSNK2A1   192 VASRYFKGPELLVDYQMYDYSLDMWSLGCMLASMIFRKEPFFHGHDNYDQLVRIAKVLGT
HsCSNK2A2   193 VASRYFKGPELLVDYQMYDYSLDMWSLGCMLASMIFRREPFFHGQDNYDQLVRIAKVLGT
SpCka1      196 VASRYFKGPELLVDFREYDYSLDIWSFGVMFAALIFKKDTFFRGRDNYDQLVKIAKVLGT
ScCKA2      203 VASRYHKGPELLVNLNQYDYSLDLWSVGCMLAAIVFKKEPFFKGSSNPDQLVKIATVLGT
ScCKA1      231 VASRFFKGPELLVDYRMYDYSLDLWSFGTMLASMIFKREPFFHGTSNTDQLVKIVKVLGT

HsCSNK2A1   252 EDLYDYIDKYNIELDPRFNDILGRHSRKRWERFVHSENQHLVSPEALDFLDKLLRYDHQS
HsCSNK2A2   253 EELYGYLKKYHIDLDPHFNDILGQHSRKRWENFIHSENRHLVSPEALDLLDKLLRYDHQQ
SpCka1      256 DELFAYVQKYQIVLDRQYDNILGQYPKRDWYSFVNRDNRSLANDEAIDLLNRLLRYDHQE
ScCKA2      263 KELLGYLGKYGLHLPSEYDNIMRDFTKKSWTHFITSETK-LAVPEVVDLIDNLLRYDHQE
ScCKA1      291 SDFEKYLLKYEITLPREFYDMDQYIRKPWHRFINDGNKHLSGNDEIIDLIDNLLRYDHQE

HsCSNK2A1   312 RLTAREAMEHPYFYTVVKDQARMGSSSMPGGSTPVSSANMMSGISSVPTPSPLGPLAGSP
HsCSNK2A2   313 RLTAKEAMEHPYFYPVVKEQSQPCADNAVLSSGLTAAR----------------------
SpCka1      316 RLTCQEAMAHPYFQVLK-------------------------------------------
ScCKA2      322 RLTAKEAMDHKFFKTKFE------------------------------------------
ScCKA1      351 RLTAKEAMGHPWFAPIREQIEK--------------------------------------

HsCSNK2A1   372 VIAAANPLGMPVPAAAGAQQ
HsCSNK2A2       --------------------
SpCka1          --------------------
ScCKA2          --------------------
ScCKA1          --------------------
```

Supplemental Figure S4

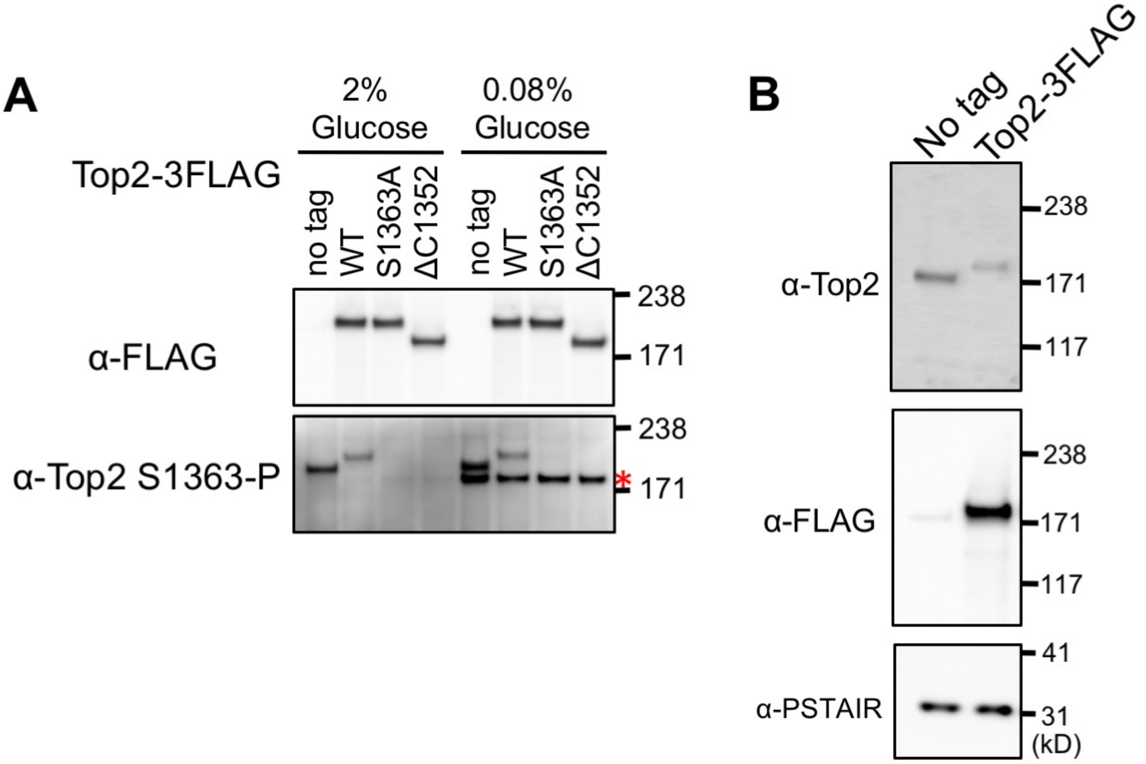

Supplemental Figure S5

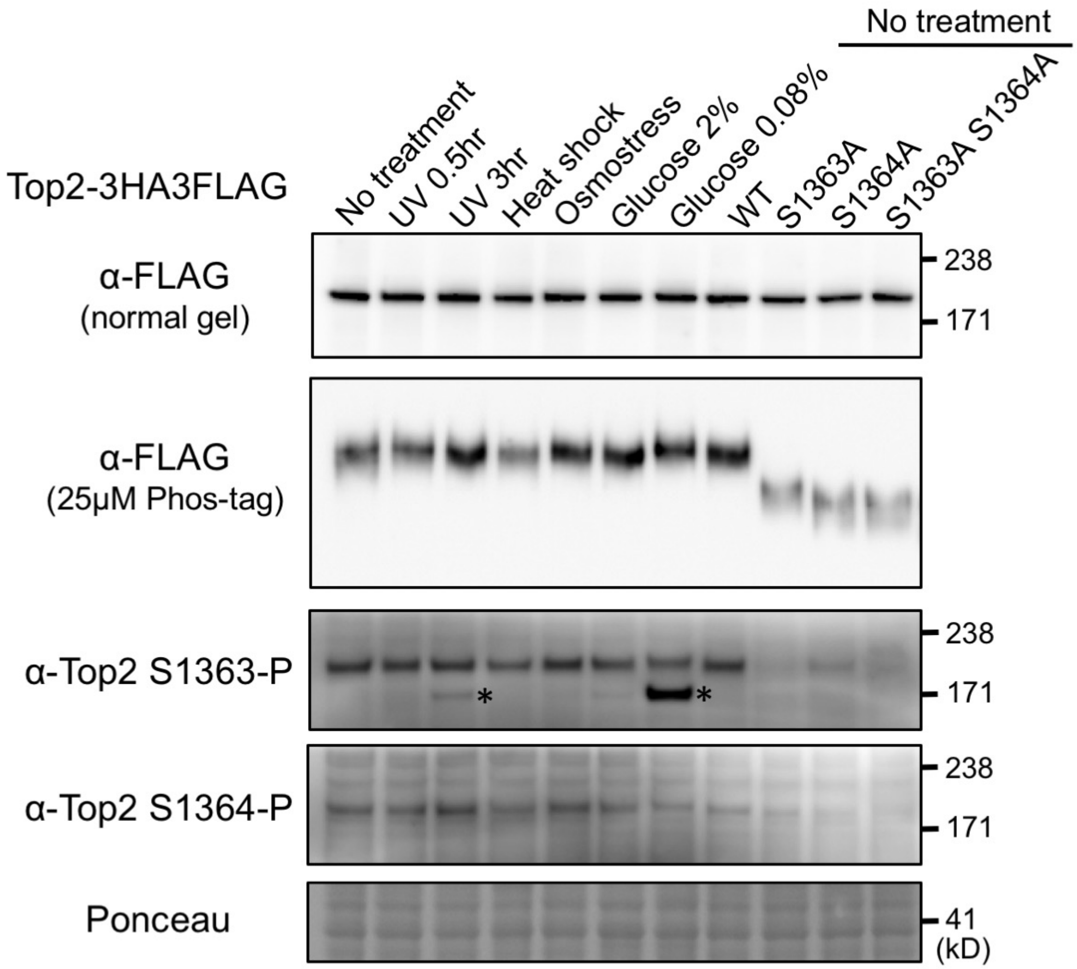

No treatment: YPD26℃
UV: 100Jm^{-2}
Heat shock: 37℃ 4hr
Osmostress: 1M Sorbitol 4hr
Glucose 2%: EMM2 +Gluc 2% 6hr
Glucose 0.08%: EMM2 +Gluc 0.08% 6hr

Supplemental Table S1. Individual data points of Top2 ATPase assay in Figure 3C

Treatment	No treatment			ICRF-193 5μM treatment		
Experiment	1st assay	2nd assay	3rd assay	1st assay	2nd assay	3rd assay
Top2 WT	0.208	0.160	0.130	0.017	0.101	0.038
Top2 2A	0.097	0.110	0.081	-0.009	0.052	-0.017

The immunoprecipitated fraction from a strain expressing an empty vector was used as a background control. Background readings were subtracted from sample readings.
